# Network Science in Egyptology

**DOI:** 10.1371/journal.pone.0050382

**Published:** 2012-11-21

**Authors:** Patrick Coulombe, Clifford Qualls, Robert Kruszynski, Andreas Nerlich, Raffaella Bianucci, Richard Harris, Christine Mermier, Otto Appenzeller

**Affiliations:** 1 Department of Psychology, University of New Mexico, Albuquerque, New Mexico, United States of America; 2 Department of Mathematics and Statistics, University of New Mexico, Albuquerque, New Mexico, United States of America; 3 Department of Earth Sciences, The Natural History Museum, London, United Kingdom; 4 Institute for Pathology, Section Paleopathology, Klinikum Műnchen-Bogenhausen, Munich, Germany; 5 Laboratory of Criminalistic Sciences, Department of Anatomy, Pharmacology and Legal Medicine, University of Turin, Turin, Italy; 6 Exercise Physiology Laboratory, University of New Mexico, Albuquerque, New Mexico, United States of America; 7 New Mexico Health Enhancement and Marathon Clinics Research Foundation, Albuquerque, New Mexico, United States of America; University of South Florida Alzheimer's Institute, United States of America

## Abstract

Egyptology relies on traditional descriptive methods. Here we show that modern, Internet-based science and statistical methods can be applied to Egyptology. Two four-thousand-year-old sarcophagi in one tomb, one within the other, with skeletal remains of a woman, gave us the opportunity to diagnose a congenital nervous system disorder in the absence of a living nervous system. The sarcophagi were discovered near Thebes, Egypt. They were well preserved and meticulously restored. The skeletal remains suggested that the woman, aged between 50 and 60 years, was Black, possibly of Nubian descent and suffered from syringobulbia, a congenital cyst in the brain stem and upper spinal cord. We employed crowd sourcing, the anonymous responses of 204 Facebook users who performed a matching task of living persons' iris color with iris color of the Udjat eyes, a decoration found on Egyptian sarcophagi, to confirm the ethnicities of the sarcophagus occupants. We used modern fMRI techniques to illustrate the putative extent of her lesion in the brain stem and upper spinal cord deduced from her skeletal remains. We compared, statistically, the right/left ratios, a non-dimensional number, of the orbit height, orbit width, malar height and the infraorbital foramena with the same measures obtained from 32 ancient skulls excavated from the Fayum, North of Thebes. We found that these ratios were significantly different in this skull indicating atrophy of cranial bones on the left. In this instance, Internet science and the use of modern neurologic research tools showed that ancient sarcophagus makers shaped and decorated their wares to fit the ethnicity of the prospective occupants of the sarcophagi. We also showed that, occasionally, human nervous system disease may be recognizable in the absence of a living nervous system.

## Introduction

Egyptology is a descriptive discipline where, normally, the details of a new find are put into appropriate context by analysis of the decorations and inscriptions found on artifacts and remains.

We hypothesized that the size of sarcophagi (ancient coffins) was fashioned to fit the prospective occupant and their decorations were adjusted to reflect the ethnicity of the deceased, if different from the ethnicity of ancient Egyptians.

Here we show that modern methods of crowd sourcing of the opinions of hundreds of Internet users, together with statistical methods, can contribute to the reliability of the interpretations of ancient finds.

Additionally, clinical paleoneurology—the examination of human nervous system function without the presence of a living nervous system—is possible only on very rare occasions [1].

We report an unusual paleoneurological opportunity on a woman who died approximately 4000 years ago in Ancient Egypt. To reach the diagnosis and place this into clinical and social context we enlisted the collective “brain power” of Facebook users and, for the first time in Egyptology, based our conclusions, in part, on “networked science” [2].

## Results

### Ancient Egyptian and modern Archeological aspects

Two rectangular sarcophagi (coffins) were excavated in 2004 near modern Luxor, Egypt, the site of the ancient metropolis of Thebes. They were found at their original burial site. The interment occurred ∼3750 years ago [3]. Unusually, therefore, the provenance of the coffins is well documented.

Their extensive decorations have been meticulously restored and together with many recovered funerary artifacts allowed the detailed reconstruction of the social aspects and life histories of the occupants of the sarcophagi [3]. Additional rare features of this find were: the smaller sarcophagus was placed within the larger one in a common tomb barely big enough to accommodate one coffin; grave robbers had broken the foot plates of both sarcophagi; and partially mummified skeletal remains were found in close vicinity to the sarcophagi.

The outer coffin was made for a judge of the Egyptian high court named Imeni; the smaller, inner coffin, for his wife named Geheset. The inscriptions and decorations allowed accurate dating to 1890-1740±150 (XII Dynasty, Middle Kingdom), before Christ years (BCE). Imeni's sarcophagus had more elaborate decorations and was made from different wood than Geheset's [3]. Based on the inscriptions and decorations of her coffin she was thought to have been one to two generations younger than her husband.

### The Udjat eye

This is a stylized, symbolic eye often found on ancient Egyptian sarcophagi. It is assumed to have been copied from a falcon's eye. This decoration is known by many names including the eye of Ra or Udjat, after the Sun God Ra, which depicts the right eye or its mirror image, the left eye, the eye of Horus, which represents the moon. These two, stylized eyes, together, encompass the entire universe. The symbol continues to be used to this day, for example the left eye as the all seeing, Masonic eye, on American banknotes.

**Figure 1:**
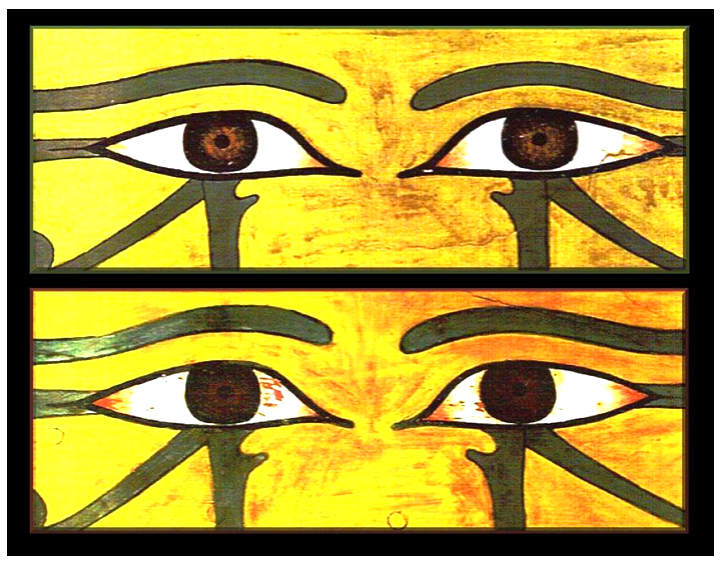
Udjat eyes from Imeni's sarcophagus (above) and Geheset's (below). Note the peri-pupilary regions of the irises of Imeni's Udjat eyes is shaded a lighter brown compared to that of Geheset's irises hinting at the different ethnicities of the sarcophagi's occupants as discerned by the decorators of the sarcophagi.

In Middle Kingdom coffins, the Udjat eyes were painted on the outer panel of the coffin and the coffins were placed facing east. The Udjat eyes on Imeni's coffin were painted on its inside, however, whereas those on Geheset's, as usual at the time, on its outside. The Udjat eyes faced each other in the burial chamber. Her mummy was placed so that her eyes were opposite the Udjat eyes presumably allowing her “to look through the two sarcophagi” using both sets of Udjat eyes to watch the sun-rise and join the sun God Ra on its daily journey across the sky. The sets of eyes on these two sarcophagi were elaborately painted and extraordinarily well preserved (Fig. 1).

### Iris color of Udjat eyes on sarcophagi

Two hundred and four anonymous people were asked, using the Internet, to pick living-people eyes that best matched the Udjat eyes for Imeni and for Geheset. Since both Imeni's and Geheset's iris color was dark we presented only dark irises for matching. We used this collective “brain power” gathered from network information provided by Facebook users in our statistical analysis of the responses to the matching tasks (see Text S1)

We found that 70.6% (P<0.001) correctly matched Geheset's iris-color on her sarcophagus' Udjat eyes with that of dark black irises of modern individuals typical of people of Black ethnicity. The matching of the lighter (but still dark) colored eyes (see Fig. 1) on Imeni's sarcophagus was 55.6% of the target number but significantly different from the 70.6% correct matches for Geheset (P<0.001) indicating that the respondents perceived Imeni's Udjat irises as different from those of Geheset's Udjat irises. This strengthens our conclusion that even the slight differences in the Udjat iris color on the two coffins were correctly interpreted by our respondents. A subset of 64/204 respondents (31.4%) also matched the irises on Imeni's sarcophagus with those of modern irises of similar color (see Fig. 1). An alternative hypothesis that respondents matched both pairs of Udjat eyes on the basis of age of the owners of the living-person eyes (in particular, to the relative smoothness of the facial skin surrounding the pairs of living-person eyes) has also been discussed in Text S1.

We determined statistically that respondents did not match randomly. Using adjustments for covariates such as age, gender, ethnicity and training in medically related disciplines or experimental format such as the order of matching of the living pictures or the presentation of only one set or of both sets of Udjat eyes simultaneously, none of these covariates altered the significant models of our results. For details of our Internet-based responses and statistical analyses see: Text S1.

We concluded that crowd sourcing, the collective matching power of 204 living human brains, using the Internet, was capable of correctly matching iris color painted on ancient sarcophagi with iris color of living people and, by extension, identifying the most likely ethnicity of the occupants of these ancient coffins.

### Skeletal remains

Anthropological examination suggested that the deceased female died between the ages of 50–60. The skull was narrow and elongated, the maxilla and mandible were significantly protruding. She was of short stature (estimated height 1.51 m) this together with her slender build, suggested that she was likely Black, and of Nubian ethnicity [3].

**Figure 2:**
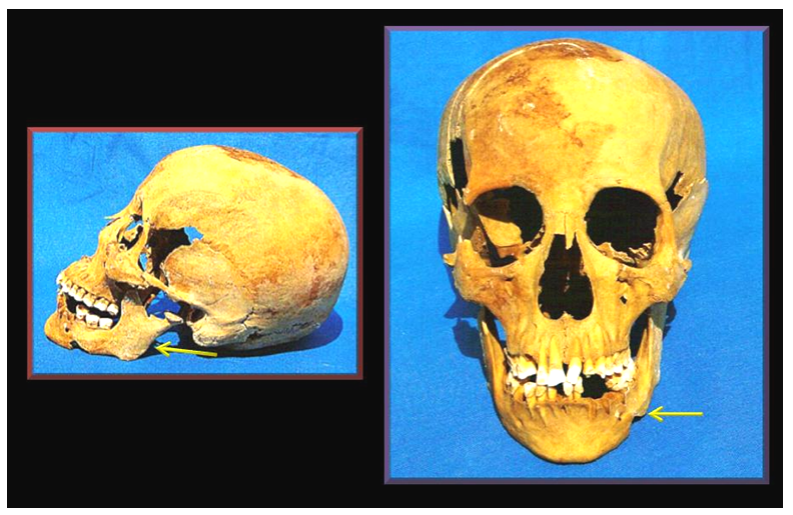
Geheset's skull. The arrows point to the hyperplastic left gonial angle. The shortened left condylar head and mushroom-like degenerate appearance of the head is shown in the lateral view.

**Figure 3:**
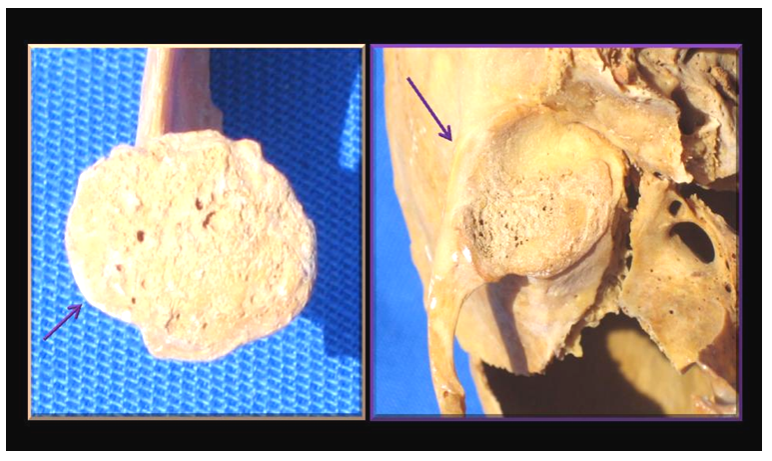
View of the joint surfaces of the left temporo-mandibular joint. The arrows point to lipping of the joint margins often seen in Charcot's joints.

**Figure 4:**
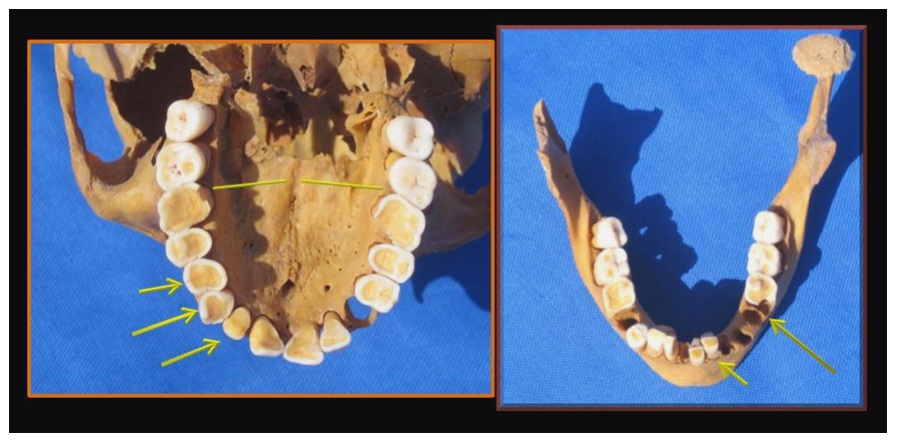
Geheset's maxillary and mandibular tooth surfaces. The arrows point to the excessive wear on the left as compared to the right side. The lines on the hard palate show the narrower left hard palate also indicating the site of measurements labeled “genial tuberosities” in table 1.

The most remarkable features of the skull included the smaller left orbit, a narrower left hard palate (Table 1) and the hyperplastic left gonial angle (Fig. 2). The left condylar head was shortened and splayed-out in mushroom-like fashion (Fig. 3) with marked degenerative changes on its joint surface at the base of the skull. The narrowing of the hard palate was matched by appropriate asymmetry of the mandible (Fig. 4 and Table 1).

**Table 1:** Measurements obtained from skull images.

Site	Right	Left	Ratio R/L
Orbit height	21	18	1.16
Orbit width	20	18	1.11
Infra orb For	1	2	0.5
Malar height	22	21	1.04
Zygom. width	10.5	10.5	1
Hard palate	13	11.5	1.13
Genial tuberosities	11	9.5	1.15

The R/L ratios, non-dimensional measures, clearly show that the left facial bones where smaller. A larger left infra-orbital foramen (Infra orb For) was also seen in another skull with smaller left facial bones, on the left, from ancient Egypt [1].

**Figure 5:**
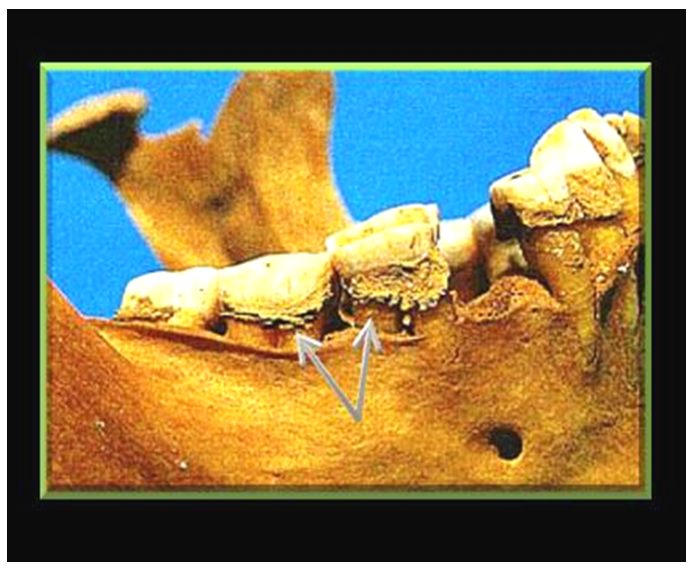
Deposition of calculus over Geheset's mandibular teeth. The arrows point to the huge amount of calculus deposition suggesting difficulty in swallowing the saliva and consequent precipitation and accretion of salivary minerals.

The teeth showed no evidence of caries but were extensively worn more on the left, the side of the degenerated joint, than the right and had abundant dental calculus which was more extensive over the mandibular teeth (Figs. 4, 5).

**Figure 6:**
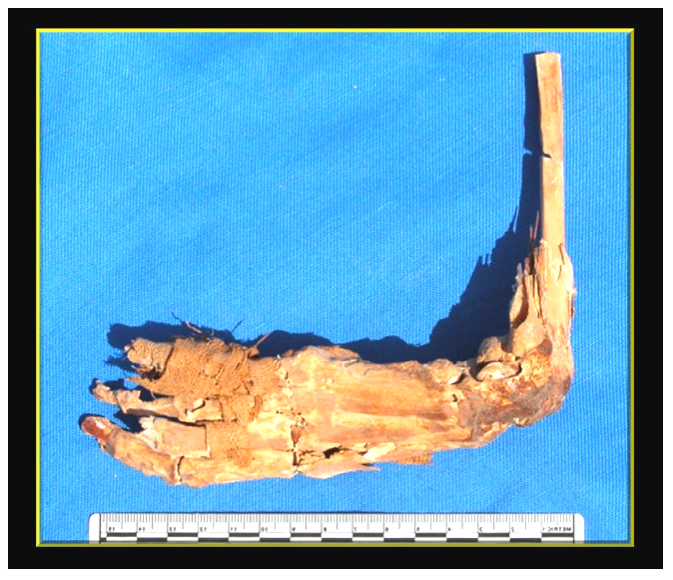
Fixed deformity of Geheset's left hand. Note the relatively well preserved soft tissues and remnants of bandages used in the embalming process.

The most remarkable skeletal features were the bilateral fixed position of the hands and the relatively well preserved soft tissues (tendons and parts of skin) and remains of bandages used in the mummification process (Fig. 6).

**Figure 7:**
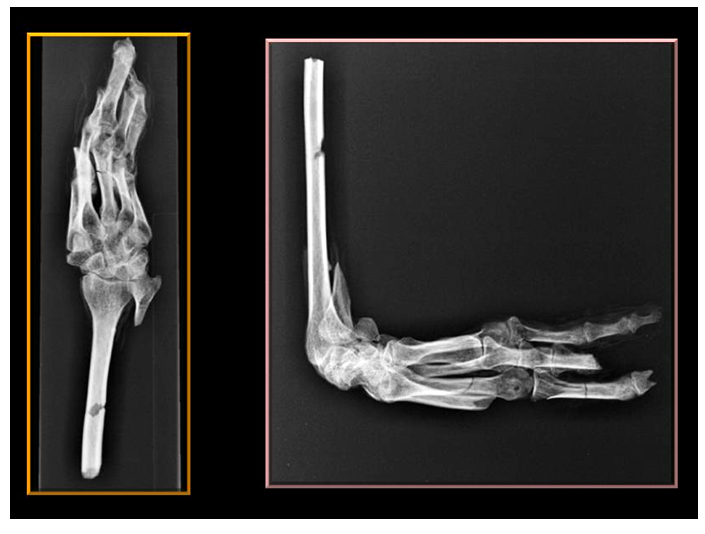
X-rays of Geheset's left hand. The images were acquired with a portable machine in the field. Several small bones show reduced density consistent with the long-standing fixed position of the deformity and disuse of the hand.

**Figure 8:**
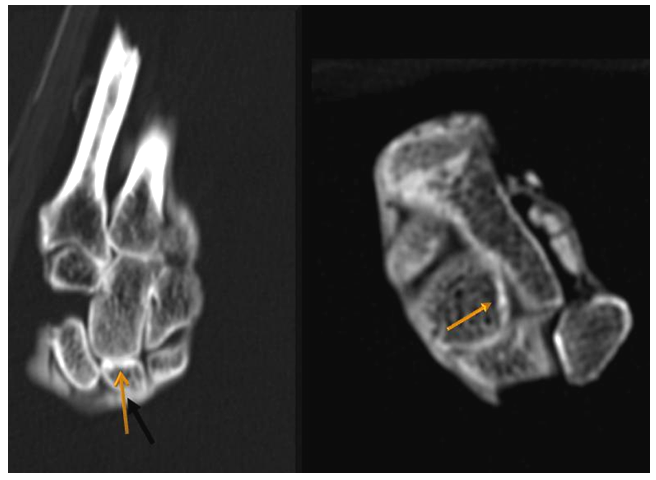
X-rays of Geheset's left hand. Minor sclerotic changes are indicated by the arrows.

The left hand was X-rayed in the field (Fig. 7) This confirmed the clinical impression of fixed long standing almost 90° flexion of the wrist joint with sclerotic changes and slight displacement of small wrist-bones (Fig. 8).

There were no discernible abnormalities in the leg bones and no evidence of joint degenerations in any of her other joints.

### Ancient neurology

Geheset's available skeletal features and the dark irises on her sarcophagus' Udjat eyes suggested she was of Black ethnicity and suffered from syringobulbia, a congenital cyst in the upper spinal cord and lower brainstem, with focal dystonia which resulted in left torticollis. Focal dystonias usually develop later in life; in this case this was likely associated with the destruction of her left temporo-mandibular joint and would have led, eventually, to the fixed position of her head-tilt towards the left shoulder evidenced by the pooling of resin in her skull after embalming.

**Figure 9:**
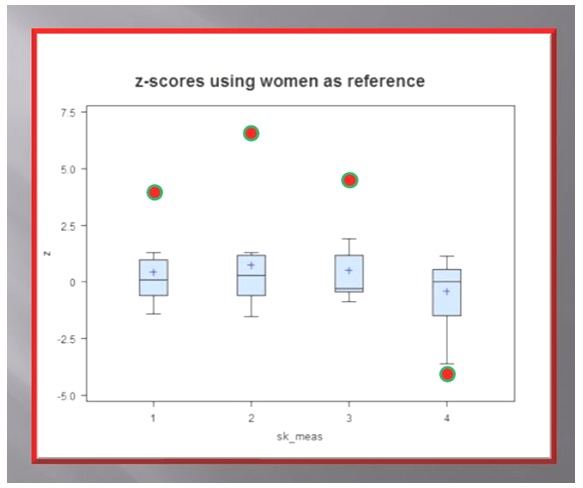
Box plots of statistical analysis of Geheset's cranial bones. The analysis was based on right/left ratios (a dimensionless measure) of her skull, obtained from photographs, to obviate the influence of possible photographic distortions, and compared to the same ratios gleaned from caliper measurements of 8 female skulls excavated from the Fayum. Geheset's ratios (red dots) are outliers with highly significant differences (z-scores) from the measures obtained from the Fayum skulls. 1 = Orbit width; 2 = Orbit height; 3 = Malar height; 4 = Infraorbital foramen. Note: the left infraorbital foramen was larger on the affected side; negative R/L ratio.

This diagnosis was based on the following localizing features: 1. The “coat hanger” like deformities of the upper limbs. 2. The absence of skeletal abnormalities in the lower limbs. 3. The gross deformity of her left temporo-mandibular joint. 4. The asymmetry of the skull bones (see Table 1 and Fig. 9) and the asymmetric wear of her teeth. 5. The pooling of embalming resin in the left parietal-temporal fossa, and 6. The increased deposition of dental calculus.

**Figure 10:**
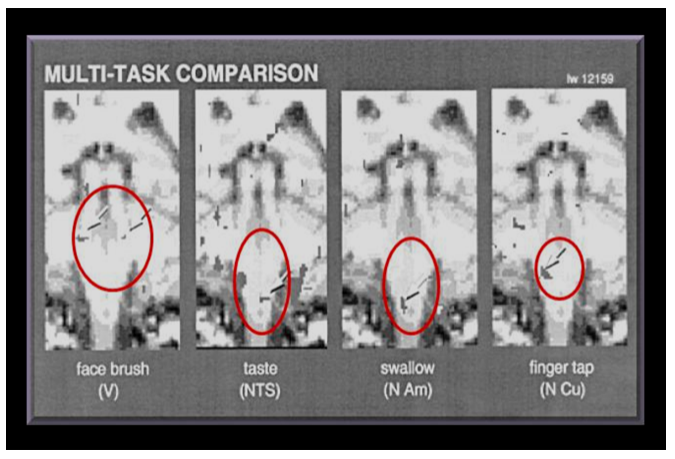
Coronal images of the brainstem regions obtained with fMRI from one normal living individual. The arrows point to the tasks used to activate various nuclei. The red ovals show the putative site of the syrinx in Geheset's brain. (*fMRI scan From: Komisaruk et al. American Journal of Neuroradiology (2002)23:609–617, with permission*. [8]).

**Figure 11:**
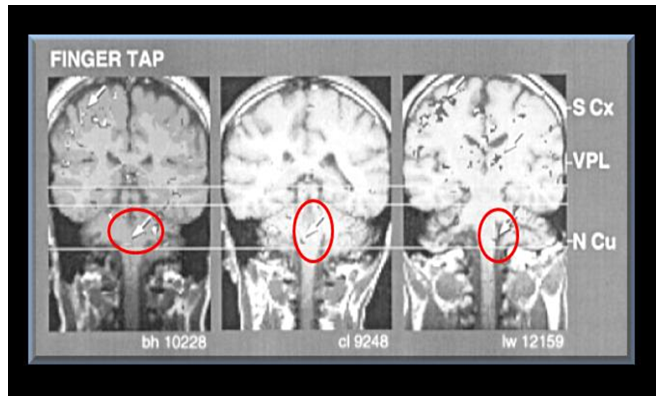
Coronal images of the brainstem regions and hemispheres obtained with fMRI from one normal living individual. The arrows point to activated regions produced by tapping the fingers against the thumb in the nucleus cuneatus (N Cu) the ventral posterior lateral nucleus (VPL) and somatosensory cortex (S Cx). The red circles show the putative site of the syrinx in Geheset's brain. (*fMRI scan From: Komisaruk et al. American Journal of Neuroradiology (2002)23:609–617 with permission*. [8]).

**Figure 12:**
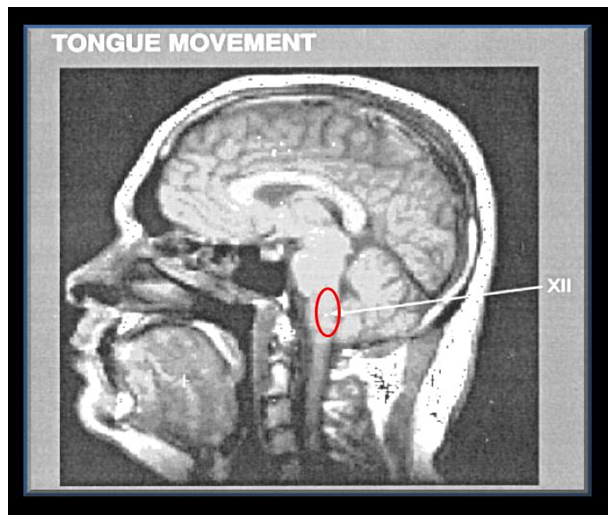
Saggital image through the tongue, the midline of the brainstem, upper spinal cord and hemispheres obtained with fMRI from one normal living individual. Activation is shown in the hypoglossal nucleus (XII) and upper segments of the spinal cord when the tongue is pushed against the hard palate. The red oval shows the putative site of the syrinx in Geheset's brain. (*fMRI* scan *From: Komisaruk et al. American Journal of Neuroradiology (2002)23:609–617 with permission*. [8]).

Because of the location of the lesion (see Figs. 10, 11, 12), its congenital nature (based on the marked asymmetry of the skull bones; see Fig. 9) and the long survival of the subject the most likely diagnosis was syringobulbia.

We next compared the caliper measurements of the orbit's width and height from 32 skulls dating to about the same period, excavated from the Fayum, North of Thebes, nearer the Nile Delta [1] with those obtained from Geheset's skull. To account for the usual variability in skull size and frequently found asymmetries we used right/left ratios of the measurements, non-dimensional numbers, for our statistical analysis (Table 1 and Fig. 9). We found that the asymmetry of Geheset's skull bones was significantly greater than the asymmetries in our available female skull bones excavated in the Fayum implying mal-development in Geheset's cranial features on the left rather than just normal human variability as found in the same region ∼4000 years ago. This was also true when Geheset's skull measures were compared to all the measurements obtained from the Fayum, males and females combined (Text S1).

### Interpretation of skeletal abnormalities

The gross deformity of her left temporo-mandibular joint (Fig. 3) is consistent with a denervated, painless joint that continued to function, possibly a Charcot's joint. Thus her trigeminal sensory nucleus function was impaired by a putative syrinx in the appropriate region of the brain stem (Figs. 10, 11, 12). Supporting this conclusion are the bilateral fixed deformities of the upper limbs and the normal lower limbs implying dysfunction of motor fibers to the fingers and because of the position of the limbs probable spastic paralysis of small muscles of the hands and the absence of any other joint degeneration or deformity (Figs. 7, 8, 9). The excessive wear of her teeth, more pronounced ipsilateral to the arthritic temporo-mandibular joint (Fig. 4) supports this interpretation because normal chewing (motor function) must have been preserved and the asymmetry of wear most likely resulted from degeneration of the left temporo-mandibular joint cartilage with consequent collapse of the joint space on the left.

To live to the age of 50–60, as Geheset did [3], and supervise a successful household, as determined from the funerary objects found in her grave [3], implies that her temporo-mandibular joint, although grossly deranged in appearance, was painless, denervated by dysfunction of the trigeminal nerve which provides sensory innervation to the joint. The preserved motor function (through the VII nerve) bilaterally is evidenced by the wear of her teeth (Fig. 4)

A congenital abnormality likely caused her disabilities because of her relatively long and successful survival, for that time-period, and the fixed position of her hands including the asymmetry of her facial bones. These could only have resulted from some lesion of the brain or upper spinal cord and the most likely site is a congenital cyst in the upper spinal cord extending into the brain stem - a syringobulbia. (Figs. 10, 11, 12).

**Figure 13:**
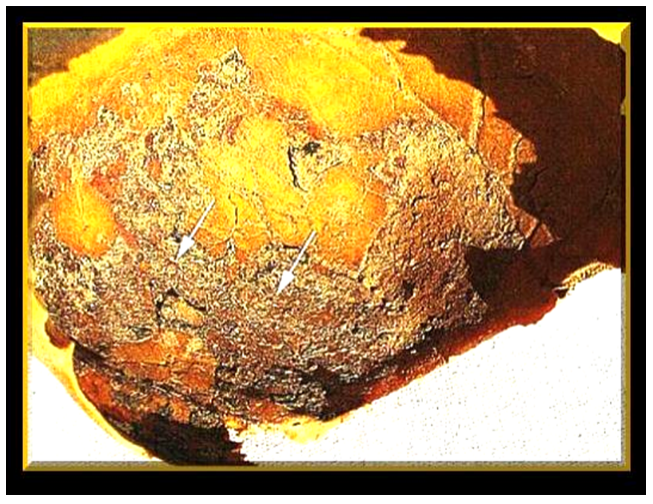
Pooling of embalming resin in the left occipital-temporal region of the calvarium. The arrows point to the resin which dried after it was poured into the empty calvarium during the embalming process.

Her head was pointing to the left shoulder when she died and was fixed in this position at the time of embalming shown by the pooling of resin in the left occipital region of her skull (Fig. 13) The best clinical explanation is that she had torticollis to the left, a focal dystonia of unknown etiology, but often found in patients with temporo-mandibular joint dysfunction. This was also consistent with her ipsilateral, degenerated temporo mandibular joint found millennia later.

While dental calculus is often more marked over the mandibular teeth, the amount of calculus deposition was extraordinarily large consistent with difficulty swallowing during life, prolonged pooling of saliva and perhaps drooling due to involvement of the nucleus ambiguous (Fig. 5) We provide a guide to the putative site of her lesion in Figs. 10, 11, 12 and Table 2.

**Table 2:** Affected brainstem nuclei in Geheset's brain.

Affected brainstem nuclei	yes/no	symptoms & signs
Trigeminal sensory (V)	yes	denervated TMJ, degenerated joint
Nucleus Cuneatus (N Cu)	yes	Finger weakness spasticity
Nucleus Ambiguus (N Am)	yes	Swallowing pooling of saliva
Nucleus Tractus Solitairus (NTS)	yes	Taste

Putative signs and symptoms caused by the life-long malfunction of these structures are indicated.

## Discussion

The extensive restorations of the elaborate decorations of this unusual find [3] and the established provenance of this unique burial gave us the opportunity to diagnose neurologic disease and postural abnormalities 4000 years after the death of the patient.

The two coffins of this unique find were entered into a small burial chamber stacked one within the other, and the putative ages of the coffins were deduced from the style of their decorations [3]. The inscriptions and elaborate hieroglyphs covering the walls of the sarcophagi led to speculations that the occupants were related to or actually royal officials at the court during the Middle Kingdom (between 1950-1750 BCE) [3].

### The color of the irises on Udjat eyes

The coffin makers fashioned their products according to the size of the occupants. Females are generally smaller than males. In this case it was evidenced by Geheset's smaller sarcophagus fitting into the larger coffin made for her husband. Close inspection of the color of the irises of the Udjat eyes on the two sarcophagi (Fig. 1) suggested that they may have also paid attention to the ethnicity of the occupants; Imeni was Egyptian and Caucasian, and Geheset was Nubian and of Black ethnicity. Although, the color of the Udjat irises on both coffins was dark, the shade of darkness of Geheset's Udjat irises was such as to suggest her Black, Nubian ethnicity.

The elaborate decorations on these sarcophagi suggested that they were especially fashioned for the prospective occupants and expensive items for the anticipated funerary rites.

### Internet-based discovery

**Figure 14:**
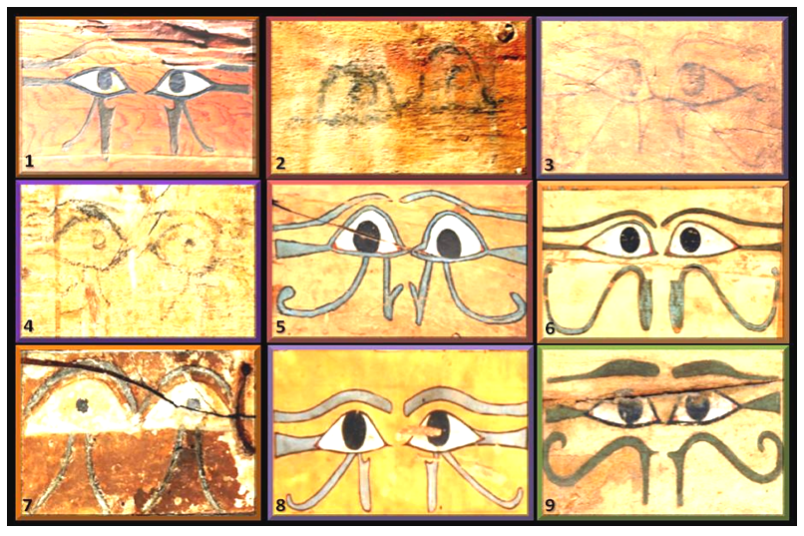
A selection of nine Udjat eyes from different sarcophagi. The sarcophagi, which date from the Third Intermediate Period and Middle Kingdom, illustrate the variations in iris coloring applied by the decorators; none were blue or green. These were presumably ordinary sarcophagi, not made especially for well paying customers, their decorations were not as elaborate as those found on Imeni's or Geheset's sarcophagi. This supports our contention that the iris colors of Imeni's and Geheset's Udjat eyes on their sarcophagi, was not standard decorators practice but they reflected, nevertheless, the prevailing iris color of Egyptians of the time. From the Egyptian Museum, Turin, Italy, with permission. 1 = First Intermediate Period (Dynasties VII–XIa from 2195 to 2066 BC). Name of the deceased: **Iti.** Sex: male Provenience: Gebelein (Inventory number: S.13719. 2 = First Intermediate Period/Middle Kingdom (Dynasties VII–XIII from 2195 to 1650 BC). Name of the deceased: unknown. Provenience: Asyut. (Iventory number: S. 7937). 3 = First Intermediate Period/Middle Kingdom (Dynasties VII–XIII from 2195 to 1650 BC). Name of the deceased: unknown Provenience: Asyut (Iventory number: S. 14428–14430). 4 = Middle Kingdom, XII Dynasty (1994- 1781 BC). Name of the deceased: unknown. Provenience: Asyut (Inventory number: Prov. 4154). 5 = Middle Kingdom, XII Dynasty (1994- 1781 BC). Name of the deceased: **Nemy** (?). Sex: female. Provenience: Asyut (Inventory number: S.08918). 6 = Middle Kingdom, XII Dynasty (1994- 1781 BC). Name of the deceased: unknown. Sex: male. Provenience: Asyut (Inventory number: S. 8807). 7 = Middle Kingdom, XII Dynasty (1994- 1781 BC). Name of the deceased: **Upuautemhat.** Sex: male. Provenience: Asyut [Inventory number: S.14459. 8 = Middle Kingdom, XII Dynasty (1994- 1781 BC). Name of the deceased: **Djefhapy.** Sex: male. Provenience: Asyut (Inventory number: S. 08876). 9 = Middle Kingdom, XII Dynasty (1994- 1781 BC). Name of the deceased: **Mereru.** Sex: male. Provenience: Asyut (Inventory number: S.08908). Egyptian chronology as given in Ikram S. (2003) [9].

To support our hypothesis we used network science [2], applied here for the first time in Egyptology. In many scientific fields, including mathematics, astronomy and particularly social sciences, discovery is now often powered by the utilization of the brains of hundreds of amateurs whose collective opinions can be analyzed statistically to reach valid conclusions [2]. The respondents' matching power and our statistical analyses confirmed that living iris color could confidently be matched with the dark irises of the Udjat eyes on Geheset's coffin (P<0.001) by naïve, anonymous respondents implying that she was of Black ethnicity and suggesting that ancient coffin-makers took notice not only of size but also ethnicity of the prospective users of their wares. To show that not all Udjat eyes were made “equal” we examined the color of the Udjat irises on nine First Intermediate period and Middle Kingdom lesser quality sarcophagi, six were dated to the XII Dynasty (1994-1781 BCE) the same period in which Geheset lived (Fig. 14). This confirmed that the iris color on Geheset's coffin Udjat eyes was not a standard used indiscriminately but most likely, as in this case, reflected the iris color during her life and that the makers and decorators of her sarcophagus were concerned not only with her small physical stature but also with her ethnicity which was strikingly different from that of her husband's.

Further analysis of the respondents' matching also revealed a subset of 64 (31.4%) of the total that chose the correct lighter colored living iris from our set of nine for Imeni's Udjat eyes and the darker living iris for Geheset's Udjat eyes and the difference in choices in this subset of respondents was also significant (P = 0.03).

Finally, additional analyses, using covariates of respondents' characteristics such as age, gender, training in medically related disciplines or in the presentation of only one or both Udjat eyes, simultaneously, for matching and order of living eyes presented for matching, showed no effect on the statistically significant results.

Thus our initial hypothesis that the color of the irises of Udjat eyes, if well preserved, might reflect, as in this case, the ethnicity of the occupants of the sarcophagi was confirmed using the modern tool of “networked science” [2].

### Skeletal abnormalities

A neurologic diagnosis in the absence of a living nervous system, paleoneurology, is only rarely attempted [1]. In this case the unusual skeletal remains discovered with Geheset's sarcophagus, however, invited several efforts to unravel the neurologic dysfunction that may have affected her during life [4], [5].

Her skull asymmetry, fixed deformities of the upper limbs with normal lower limbs and survival into middle age suggested that she may have had a non-life threatening congenital abnormality that affected the brain stem, which extended into the upper spinal cord. This conclusion was reached using the usual steps in clinical neurology such as localization of the lesion followed by attempts at guessing the functional implications and pathogenesis of the lesion(s).

The extensive left temporo mandibular joint arthropathy implied a life-long painless but still functioning joint. This is also supported by the marked wear of the teeth on both sides. Such massive joint destruction with continued function, evidenced by the even more pronounced wear of the teeth on the affected side and the hyperplastic left gonial angle, may have been promoted by the collapse of the joint, shortened condylar head and disappearance of the cartilage.

Osteoarthritis of the temporo-mandibular joint in ancient remains has been assessed [6]. The norm, at those ancient times, was that well-worn teeth had twice the incidence of osteoarthritis compared to those whose diet was more refined and who lived in more recent times such as 17–20^th^ German and British cultures. But unilateral severe osteoarthritis of the temporo-mandibular joint with a normal contra-lateral joint, as found in Geheset, has not previously been reported in three hundred and forty-eight ancient cranial remains [6]. This is consistent with our interpretation that this joint destruction (see Fig. 4) most likely represents a Charcot joint which is known to function painlessly and which represents, in this case, loss of trigeminal nerve (sensory) innervation to the left temporo-mandibular joint.

The remarkably extensive deposition of calculus [7] was most likely the result of impaired swallowing and pooling of the saliva suggesting hypoglossal (XII) nuclear dysfunction.

The dental health of 93 Egyptians excavated at Amarna from the same historic period was reviewed [8]. Like Geheset they had no caries and extensive dental wear but, importantly, very little calculus deposition. Geheset's remarkable calculus could therefore be ascribed to the relatively long pooling of saliva in her mouth with precipitation of salivary constituents resulting from impairment of her swallowing function. Swallowing is related to hypoglossal nerve function and activation of this nucleus can be seen on pushing the tongue against the hard palate, a necessary act during swallowing (see Fig. 12). Thus the putative lesion must have extended at least to the lower end of the spinal nucleus of the trigeminal nerve and to the level of the hypoglossal nucleus.

The fixed positions of her hands are reminiscent of the position assumed by tapping fingers against the thumb and consistent with lesions affecting the nucleus cuneatus bilaterally (see Fig. 11). This interpretation assumes that supranuclear lesions lead to spasticity and the eventual immobility of the wrists.

Taken together, these skeletal features point to a long-standing disorder affecting the nuclear structures in the brain stem and the upper spinal cord, most likely a congenital cyst—a syrinx.

To reach this diagnosis we show that the traditional techniques used in clinical neurology, that is, localization of the lesion based on clinical deficits and guessing at pathogenesis continued to serve well but they may have been overshadowed, in our study, by modern techniques such as fMRI and crowd sourcing.

## Methods

### Internet-aided Egyptology

The study was approved by the Human Research Protections Office of the University of New Mexico Albuquerque NM, USA, Protocol #11-613 dated 26-January-2012.

### Online experiment

Participants accessed the online experiment through any browser on any computer or device connected to the Internet, at the following address: http://www.patrickcoulombe.com/udjat.

For details of the Internet-conducted interrogation of respondents (Facebook users) see Text S2

### Statistical analyses of Internet generated responses

For details of the analyses see Text S1.

Briefly, there were three binary factors that could be determined for the nine pictures of living eyes and that differed for Imeni and Geheset: there were seven males and two females; four pictures had somewhat lighter colored dark irises consistent with Caucasian ethnicity (Egyptian) and five had dark irises more like a person of Black ethnicity (Geheset).

We tested whether respondents correctly matched more than expected by random choice by a one sample, two-tailed binomial test, comparing the observed percentage to the random choice percentage.

Additionally, we tested whether the matches were random and found, using a Chi-squared goodness-of-fit for the 9×9 = 81 combined choices of the living eyes that they were not random. Lastly, we used Fisher's exact test for the 9×9 frequency table and the paired responses were not independent of each other.

We compared the right/left ratios, a non-dimensional measure, obtained from photographs of Geheset's skull with similar measures using calipers on thirty two skulls excavated from the Fayum, now kept at the Natural History museum, London, UK. We used the measures obtained from the height and width of the orbits, the malar height and the width of the infraorbital foramena for comparison purposes.

### Paleoneurology

Standard neurological diagnostic techniques were applied. Because of the absence of a living nervous system we used the available clues from the skeletal abnormalities of Geheset's remains to deduce the site of the lesion. We then, diagrammatically, delineated the extent of the lesion on the fMRI images obtained from living normal humans from reference [8].

### fMRI of living brain

Standard **f**unctional **MR I**maging (fMRI) **b**lood **o**xygen **l**evel-**d**ependent (BOLD) techniques were used. The normal subject performed tasks to activate the motor and sensory nuclei of the brainstem fully describe in reference [8]. The results were analyzed using accepted statistical techniques [8].

This widely used, non-invasive research method, employs neuronal activity induced by appropriate tasks, given to living subjects, to alter the hemodynamic pattern in the neural structures normally involved in these tasks. This in turn produces alterations in functional magnetic resonance imaging (fMRI) signals which can be recorded. The activated structures are detected by the changes in **b**lood-**o**xygen-**l**evel-**d**ependent (BOLD) signal intensity induced by the neuronal activity. To give a visual representation of the structures which must have been affected in Geheset's brain during her life some 4000 years ago, while doing ordinary activities such as swallowing or tapping of fingers against the thumb, we used (fMRI) images to graphically illustrate the site of the lesion which likely produced the skeletal changes (see Figs. 10, 11, 12).

## Supporting Information

Text S1 **Statistical analyses.**
(DOCX)

Text S2 **Online information.**
(DOCX)
