# Supplementary material for: Network Science in Egyptology
Source: PLoS One. 2012 Nov 21;7(11):e50382. doi: 10.1371/journal.pone.0050382 (PMC3503959; doi:10.1371/journal.pone.0050382)
Supplement: Text S1 — Statistical analyses. (DOCX) [file pone.0050382.s001.docx]

**Statistical Analysis**

In line with our hypothesis, there are 3 binary factors that can be determined for the 9 pictures of eyes and that differ for Imeni and Geheset. Imeni was male, Egyptian, of Caucasian ethnicity and the Udjat eyes on his sarcophagus had lighter but still black colored irises, whereas Geheset was female, Nubian, of black ethnicity and had darker black irises in the Udjat eyes on her sarcophagus. In the 9 numbered pictures of living eyes male=(#2,#3,#4,#5,#6,#7,#8 ), Andeans (Caucasian ethnicity) = (#3,#5,#7,#8), and lighter colored irises = (#3,#5,#7,#9). Whether respondents correctly matched each of these factors more than expected by random choice is tested by a one-sample, two-tailed binomial test comparing the observed percentage to the random choice target percentage. The analysis for age (younger vs. older) among the 9 pictures of living eyes is not as straight forward.

In case our simple hypothesis proves correct. It is also important to know whether the matches made for Imeni and Geheset were: 1) completely at random, 2) independent choice, and/or, 3) differed for the two sets of Udjat eyes. A Chi-squared goodness-of-fit for the 9x9 =81 combined choice was used to test whether choices are completely at random and Fisher’s exact tests for the 9x9 frequency table of paired choices will be used to test independence of the choices. Both tests need to be exact tests because of the low frequencies in many of the cells and we use the Monte Carlo simulation option with 1,000,000 simulations in Statxact version 9.0 as a feasible exact computation. We use residuals (O-E) /$\sqrt{E}$ where O=frequency and E=expected frequency under independence to identify where choice for Imeni’s and Geheset’s Udjat eyes might differ. These differences will be tested (mostly by McNemar’s test) to determine significance. The respondents provided information (gender, ethnicity, age, training in medically related fields,) that will be used as covariates in the above analyses. In the database of 204 respondents, a small percentage did not provide the following demographic information: age=1%; ethnicity=2.9%; gender=2.5%; and training=1.5%. In addition, there is information about the experiment (random order of the presentation of Imeni and Geheset Udjat eyes, as well as the format (one set of Udjat eyes per page verses both sets on one page).

|  | |
| --- | --- |
|  |  |
| Ethnicity | Frequency/% |
| Asian/Oriental | 8 ( 4.0) |
| Black | 10 ( 5.0) |
| Caucasian | 155 (77.9) |
| Hispanic | 25 (12.6) |
| Native American | 1 ( 0.5) |
| Gender |  |
| Female | 118 (59.1) |
| Male | 81 (40.9) |
| Age |  |
| 18-29 | 51 ( 25.3) |
| 30-39 | 43 (21.3) |
| 40-49 | 38 (18.8) |
| 50-59 | 42 (20.8) |
| 60-69 | 17 ( 8.4) |
| 70+ | 11 ( 5.5) |
| Training in Medical Fields | |
| No | 101 (50.3) |
| Yes | 100 (49.7) |

Summary demographic and design information is given in **Table 1.** The 204 respondents reported an approximate median age of 40 and 50% of them had some specialized training; and typically reported being female and Caucasian.

| **Table 2** | |  |  |  |  |  |
| --- | --- | --- | --- | --- | --- | --- |
| Imeni | Frequency | PERCENT | Geheset | Frequency | PERCENT | McNemar P-value |
| 1 | 36 | 17.6 | 1 | 48 | 23.5 | 0.15 |
| 2 | 15 | 7.4 | 2 | 18 | 8.8 | 0.49 |
| 3 | 5 | 2.5 | 3 | 6 | 2.9 | 0.71 |
| 4 | 34 | 16.7 | 4 | 38 | 18.6 | 0.55 |
| 5 | 9 | 4.4 | 5 | 1 | 0.5 | 0.005 |
| 6 | 29 | 14.2 | 6 | 39 | 19.1 | 0.13 |
| 7 | 7 | 3.4 | 7 | 4 | 2.0 | 0.32 |
| 8 | 3 | 1.5 | 8 | 1 | 0.5 | 0.32 |
| 9 | 66 | 32.4 | 9 | 49 | 24.0 | 0.0496 |
|  | 204 | 100 |  | 204 | 100 |  |

For Imeni’s Udjat eyes, the respondents did not correctly match Imeni’s ethnicity (24.5% correct versus a target percent of 44.4%, binomial test, P<0.001, but in the wrong direction); Imeni’s gender (50.0% correct versus a target percent of 77.8%, binomial test, P<0.001 but in the wrong direction); or his lighter colored irises (42.7% correct vs 44.4% target, P=0.67). For Geheset’s Udjat eyes, the respondents did not correctly match Geheset’s ethnicity (51.0% correct versus a target percent of 55.6%, binomial test, P=0.21); but they did match her dark Udjat irises 70.6% correct vs 55.6% target, P<0.001). None-the-less, the respondents did choose differently for Imeni than they did for Geheset (McNemar, P<0.001). They did also match her gender correctly (47.6% correct vs 22.2% target, P<0.001); however, this is not totally convincing, since for Imeni’s Udjat eyes, respondents also matched to the female pictures 50% of the time (no difference, McNemar, P=0.43). Concerning age of the living eyes, the choices for both Imeni and Geheset were the younger pictures (88.2% and 94.1%, respectively) more than the target 55.6%, but there was no difference in these choices between them (McNemar, P=0.34) .

Having obtained a verification for one of our simple hypotheses (correct match for Gehest’s iris color), we turned to answering other questions. First, respondents did not match pictures at random; the choices of each of the 9 pictures are clearly not uniform (Table 2). Note that the choices for pictures #5 and #9 are different for Imeni and Geheset, but we have other evidence of differences.

Secondly, we use Fisher’s exact test for independence (with 1,000,000 Monte Carlo simulations to make the test for a 9x9 contingency table tractable) to compare between the choices for Imeni and Geheset.

The choices were dependent (not independent, P<0.001). Third, we use the residuals (O-E) /√E in Fig 1.

Residuals are greater than 2 for the paired choices of pictures (Imeni, Geheset) = (1,9), (2,2),(3,3), (4,4),(5,5),(6,6), and (7,7). Our interest is in when the paired choices differ, namely for (1,9). Other pairs of different choices that are higher than expected under independence are (7,1), (8,9) and (9,1).

Since pictures 7 and 8 are very low frequency for both Imeni and Geheset, we are focusing on (1,9) and (9,1). Pictures 1 and 9 are young black females (the only females among the 9 pictures); a difference is that #9 has darker irises and #1 has lighter irises. So, for the subset of 64 of 204 (31.4%) respondents that choose the pairs (1,9) or (9,1), 45 (70.3%) choose the correct lighter iris color (picture #1) for Imeni and 32 (50%) choose the correct dark iris (picture #9) for Geheset. And these choices differed (McNemar, P=0.03).

An equal number, 64 (31.4%) of 204, chose the same picture for both Imeni and Geheset.

The choice of picture #5 in table 2 also differed for Imeni and Geheset (McNemar, P=0.005), which also supports that the correct Udjat iris color was chosen for Imeni’s Udjat eyes.

Analysis with covariates. The respondents’ choice (31%) of the same picture for both Imeni and Geheset was not related to any respondent characteristic (age, gender, training) nor to experimental design factors (order, format). Adjustment for all of these covariates in the statistically significant models above did not alter those results.

Conclusion. The simple hypothesis that respondents correctly matched Geheset’s Udjat iris color was verified, and the choice of Udjat iris color differed for Imeni and Geheset. Respondents chose some pictures over others, namely younger pictures. In a subset (31%), both choices were the (only) two females; in this subset, the choices of #1 or #9 differed for Imeni and Geheset with correct Udjat iris color being chosen for Imeni 70.3%. However in another subset (31%), respondents made exactly the same choice for Imeni and Geheset Udjat iris color.

**Statistical Comparison of Geheset’s skull to the skulls excavated at Fayum, Northern Egypt**

Using measurements of skulls from the Fayum as reference measures, we computed z-scores (measured-mean)/standard deviation). Z-scores whose absolute values are greater than 1.96 are considered to be significantly different from the reference (a z-test). In the paper (Fig. 10) we use the female skulls as reference; here we use the pooled men and women skulls as reference.

**Fig. 1 Pooled measurements (ratios) of men and women combined obtained from the Fayum skulls.**

The analysis was based on right/left ratios (a dimensionless measure) of Geheset’s skull, obtained from photographs, to obviate the influence of possible photographic distortions, and compared to the same ratios gleaned from caliper measurements of 8 female skulls and 24 male skulls excavated from the Fayum. Geheset’s ratios (red dots) are outliers with highly significant differences (z-scores) from the measures obtained from the Fayum skulls. 1=Orbit width; 2=Orbit height; 3=Malar height; 4=Infraorbital foramen. Note: the left infraorbital foramen was larger on the affected side; negative R/L ratio.
