# Supplementary material for: Network Science in Egyptology
Source: PLoS One. 2012 Nov 21;7(11):e50382. doi: 10.1371/journal.pone.0050382 (PMC3503959; doi:10.1371/journal.pone.0050382)
Supplement: Text S2 — Online information. (DOCX) [file pone.0050382.s002.docx]

**On line experiment carried out on Facebook users.**

Participants accessed the online experiment through any browser on any computer or device connected to the Internet, at the following address: <http://www.patrickcoulombe.com/udjat>

The top of the page showed the following introduction: “Udjat eyes are stylized eyes often found in the decorations of ancient Egyptian sarcophagi (coffins). The eyes presented in this study are from two different sarcophagi that are about 4,000 years old. You must be at least 18 years old to participate.” And: "Please click on the picture of the human eyes from the right column that matches best the painting of the Udjat eyes from the left column."

Additionally, the recruitment message included the following:

“After accessing the web page you will be asked to click a box that you are consenting to participate in this research. Once you’ve agreed to participate, you will be asked to match two 4000-year-old stylized pictures of eyes from Egyptian sarcophagi (coffins) with one or two of nine pictures of living human eyes. Each matching task will take approximately 60 seconds, for a total of approximately 120 seconds (2 minutes). At the end of the matching task you will be asked to provide some personal information such as age group, ethnicity, gender, and current or past training in medical or other health sciences. Answering these personal questions is not mandatory for completion of the study. All your responses will be kept anonymous and confidential.”

Participants were randomly assigned to either have the two matching tasks on the same page or on two different pages. If the two matching tasks were on two different pages, when the participants clicked on one of the human eyes from the right column, the picture of the Udjat eyes in the left column was replaced with the second picture of Udjat eyes and the positions of the 9 human eyes in the 3x3 grid of the right column were randomly changed. For the second matching task, the statements at the top of the page remained the same. When the participants clicked on one of the human eyes, they were then brought to the final page of the experiment.

Conversely, if the two matching tasks were on the same page (one above the other, as in Fig. 2), when the participant clicked on one of the human eyes from the right column for a particular picture of Udjat eyes from the left column, the background of the picture of the human eyes that had been clicked became grey, to indicate that this picture had been chosen as the participant’s answer. If they clicked on the same picture again, the background was set back to the default color (white). Participants were free to change their answers any number of times. Below the two matching tasks, a button read “Continue”, which when clicked brought the participants to the final page of the experiment. If no picture of human eyes had been selected to match either one or both of the pictures of Udjat eyes, then an alert was displayed saying “You must select a picture of human eyes for each of the two pictures of Udjat eyes in order to be able to continue.”

Which one of the two different Udjat eyes was shown first and which one second was determined randomly by the webpage, regardless of whether the two matching tasks were on the same page or on different pages. Also, the position of each set of human eyes in the 3x3 grid of the right column was determined randomly for both matching tasks.

After participants completed the two matching tasks, they were brought to the final page of the experiment. This page stated at the top: "Please tell us more about yourself. If you do not want to answer any or all of the questions, please leave these questions blank. After you are done, click on 'I am done with this experiment'." Below this statement, five different questions on five separate lines appeared. On the first line, the participant read "My age group is:" with the option of choosing either 18-29, 30-39, 40-49, 50-59, 60-69, 70 or more, or a blank choice. The default option was blank. On the second line, the participant read "I identify with this ethnicity:" with the option of choosing Caucasian, Black, Hispanic, Asian/Oriental, Native American, or a blank choice. The default option was blank. On the third line, the participant read “My gender is:” with the option of choosing Male, Female, or a blank choice. The default option was blank. On the fourth line, the participant read "Have you taken, or are you currently taking, training in the medical or other health sciences?" with the option of choosing Yes, No, or a blank choice. The default option was blank. Finally, on the fifth line, the participant read “I confirm that I am at least 18 years old.” with the option of either checking this option or leaving it blank. By default the checkbox was not checked.

At the bottom of this page, there was a button that said "I am done with this experiment". If the participant had not checked the statement “I confirm that I am at least 18 years old”, an alert was displayed notifying the participant that “You must confirm that you are at least 18 years old in order to send your responses.” If the participant used a browser that did not allow for such a verification to be made, then the server itself made the verification: If the participant had not checked the statement confirming their adult age, the questionnaire was reloaded with the participant’s answers already entered, with the warning “You must confirm that you are at least 18 years old in order to send your responses” printed directly on the page, in red, next to the statement “I confirm that I am at least 18 years old”. Therefore, regardless of what specific application the participant used to reach the website of the study, the responses could not be sent without the participant confirming their adult age. Once the participant had checked that statement and clicked the button “I am done with this experiment”, the website brought the participant to a page that stated: “Thank you for taking part in this experiment. Your responses are completely anonymous and confidential. Furthermore, no personal or identifiable information from your computer was collected”.

When the button was clicked, the participant's responses to the first matching task, to the second matching task, and to the five demographic questions were automatically stored in a database hosted on a professional (paid) and secured server, that was created exclusively and solely for use in this experiment, and that is protected by a password known only to the research team. Each participant was assigned a unique identifier number in sequence. No other information was sent or collected, whether from the participants themselves, their browser, their Internet connection, or their computer.
